# Limited evidence on the use of fibrin clot as an adjunct to meniscal repair with sutures: A systematic review

**DOI:** 10.1002/jeo2.70503

**Published:** 2025-10-31

**Authors:** Laura Amaral Coelho de Azevedo, Rayanne Carneiro Torres de Novaes, Gabriela de Paula Leite Rocha Alcântara Del Campo, Maria Fernanda Pereira Junqueira, Diego Escudeiro de Oliveira, Pedro Baches Jorge

**Affiliations:** ^1^ Santa Casa de Misericórdia de São Paulo São Paulo Brazil; ^2^ Hospital Sírio Libanês São Paulo Brazil

**Keywords:** biological augmentation, fibrin clot, knee injuries, knee surgery, meniscal repair

## Abstract

**Purpose:**

Meniscal tears are common knee injuries that often require surgical repair due to pain and mechanical symptoms. Limited vascularisation in specific meniscal regions hinders healing, encouraging the use of fibrin clots as a biological augmentation strategy. This study aims to critically evaluate the available evidence on the use of fibrin clot as a biological adjunct in meniscal repair with sutures and its potential impact on healing outcomes.

**Methods:**

A systematic review was conducted following the PRISMA guidelines, encompassing the PubMed, Embase and Scopus databases up to April 2025. Studies investigating meniscal repair augmented with a fibrin clot were included in the analysis. Data extracted included study design, type of meniscal lesion, surgical technique, clot preparation and failure rates.

**Results:**

Eleven studies were included, comprising both retrospective case series and limited comparative studies. Most studies involved small sample sizes and heterogeneous populations, characterised by diverse meniscal tear patterns, surgical techniques and clot sources. Reported healing rates ranged from 43% to above 90%, but outcome definitions were inconsistent. A critical risk of bias was identified in most of the studies.

**Conclusions:**

The current evidence on the use of fibrin clot as an adjunct to meniscal repair is limited and inconsistent. Although some studies suggest potential benefits, the overall quality of evidence is low. Further high‐quality randomised controlled trials are needed to determine whether fibrin clot improves meniscal healing outcomes.

**Level of Evidence:**

Level IV, systematic review.

**Clinical Trail Registration:**

This systematic review was registered in PROSPERO (registration number: CRD42024614021).

**Abbreviations**
ACL: anterior cruciate ligament
IKDC: International Knee Documentation Committee
MRI: magnetic resonance imaging
NIH: national institutes of health
PRISMA: preferred reporting items for systematic reviews and meta‐analyses
PROMs: patient‐reported outcome measures
PRP: platelet‐rich plasma
RCT: randomised controlled trial

## INTRODUCTION

Meniscal tears are among the most common knee injuries and are increasingly managed with repair rather than meniscectomy to preserve joint biomechanics and delay degenerative changes [7, 23]. These injuries are broadly classified as degenerative or traumatic, with distinct pathophysiology, prognosis and treatment implications. Degenerative tears, more prevalent in older patients, are often horizontal, complex, or flap‐type, typically involving the avascular white–white zone, which has limited intrinsic healing potential [9, 18]. Traumatic tears usually occur in younger, active individuals after acute knee trauma, frequently in association with anterior cruciate ligament (ACL) injuries. These lesions are often vertical longitudinal, radial, or bucket‐handle, located in the vascularises red–red or red–white zones, where healing potential is greater [6, 16].

Despite advances in surgical techniques, meniscal repair carries a substantial risk of failure. A recent meta‐analysis of 3829 repairs reported an overall failure rate of 14.8%, with higher rates in medial repairs and in knees without concomitant ACL reconstruction [22]. Long‐term follow‐up studies show that failures may occur years after surgery, with rates approaching 19% at 7 years [23]. Tear pattern, location, chronicity and joint instability are key prognostic factors, with radial, horizontal, degenerative and white–white zone lesions showing consistently higher failure rates than peripheral longitudinal tears in the red–red zone [7, 16].

Given that meniscal healing is highly dependent on vascular supply, biological augmentation has emerged as a strategy to enhance repair outcomes [27, 28]. Autologous fibrin clot implantation at the repair site is a low‐cost, technically simple method that serves as a scaffold rich in growth factors, supporting cell adhesion, promoting angiogenesis and facilitating tissue integration [4, 19]. First described by Arnoczky et al. in 1988 for avascular meniscal zones [1], this technique has gained renewed interest, particularly for complex or avascular tears. However, the current literature consists mainly of small, retrospective series with heterogeneous methodologies, and high‐quality comparative data are lacking.

The clinical benefit of fibrin clot augmentation remains uncertain, especially regarding clinically or arthroscopically confirmed healing rates and failure rates. Therefore, this systematic review aims to critically appraise and synthesise the available evidence on fibrin clot use as an adjunct to meniscal repair, focusing on these outcomes and the methodological quality of the studies. We hypothesise that fibrin clot augmentation is associated with improved healing rates and reduced failure rates, particularly in tears with limited intrinsic healing potential.

## METHODOLOGY

This systematic review followed the PRISMA (preferred reporting items for systematic reviews and meta‐analyses) guidelines and was registered in the PROSPERO database. The objective was to critically assess the efficacy of fibrin clots as a biological adjunct to suture‐based meniscal repair, with a particular focus on evaluating clinical outcomes, including healing rates, failure rates and functional recovery.

### Eligibility criteria

Inclusion criteria comprised randomised controlled trials (RCTs), nonrandomised controlled studies, cohort studies and case series involving patients of any age who underwent meniscal repair with fibrin clot augmentation. Studies were included if they reported clinical outcomes, specifically: (1) healing rates confirmed through clinical examination, arthroscopy and/or magnetic resonance imaging (MRI); (2) failure rates, defined as the need for reoperation, re‐tear, or persistent symptoms; and (3) validated functional outcome scores, such as the International Knee Documentation Committee (IKDC) score and the Lysholm score.

Exclusion criteria included case reports, narrative or systematic reviews and studies involving meniscal repair without fibrin clot augmentation.

### Search strategy

A comprehensive search was conducted in PubMed, Embase and Scopus databases for studies published up to April 2025. The search strategy applied the following Boolean string across databases: (‘fibrin clot’ OR ‘blood clot’ OR ‘fibrin glue’) AND (meniscus OR meniscal) AND (repair OR suture).

### Study selection and data extraction

Two independent reviewers screened titles, abstracts and full‐text articles using the predefined inclusion and exclusion criteria. Any disagreements were resolved through discussion with a third reviewer. Data extraction was conducted using a standardised form to collect key variables, including authorship, country of origin, year of publication and study design. Additional data included patient demographics, meniscal tear characteristics, suture repair techniques and methods of fibrin clot application. All steps were conducted independently by two reviewers, with conflicts resolved through consensus or consultation with a third reviewer.

### Data synthesis

**Figure 1:**
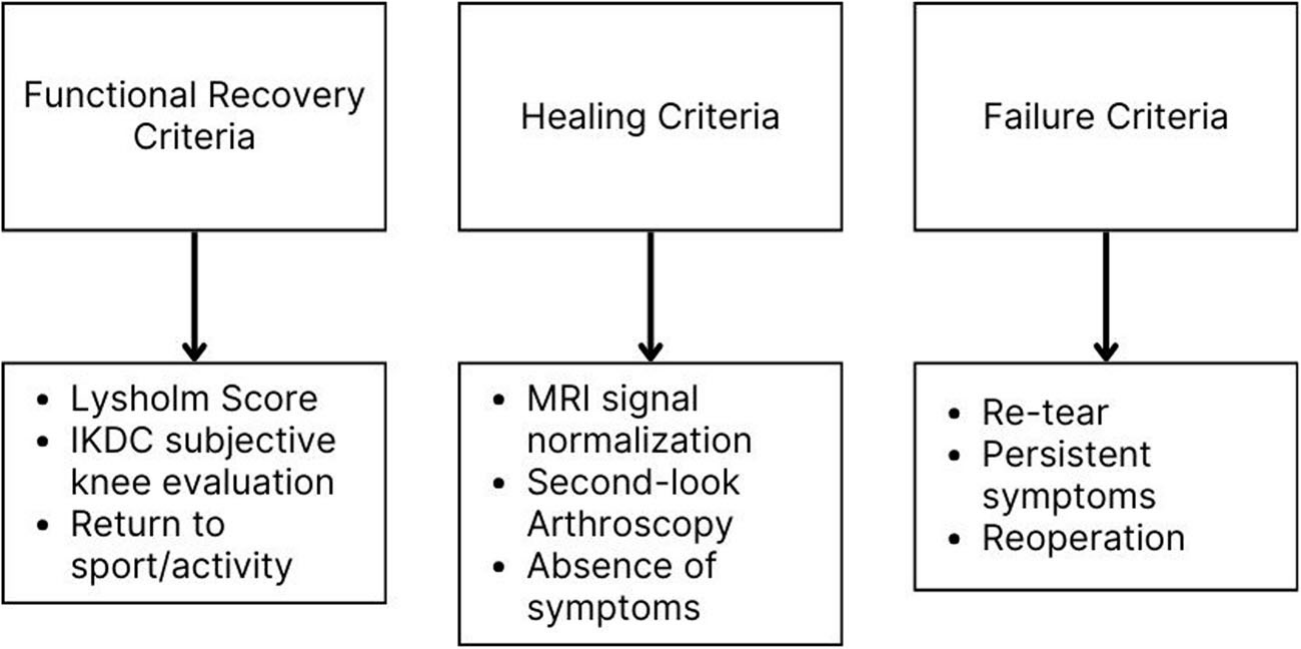
Criteria for clinical assessment. Overview of clinical criteria commonly employed to assess functional recovery, tissue healing and surgical failure following meniscal repair. IKDC, International Knee Documentation Committee; MRI, magnetic resonance imaging.

The primary outcomes analysed were healing rates, failure rates and functional recovery. Due to substantial heterogeneity in study design, patient populations, lesion types and locations, surgical approaches, outcome definitions and follow‐up durations, a quantitative meta‐analysis was not feasible. Accordingly, a qualitative descriptive synthesis was performed to provide an integrated overview of the potential impact of fibrin clot augmentation on meniscal repair outcomes across different clinical scenarios. Figure 1 summarises the most frequently used clinical criteria to assess the primary outcomes.

### Risk of bias assessment

The risk of bias was assessed using the Cochrane Risk of Bias 2 (RoB 2) tool for RCTs, the ROBINS‐I tool for nonrandomised studies, and the National Institutes of Health (NIH) quality assessment tool for case series. Studies were categorised as having low, moderate, or high risk of bias, and this classification guided the interpretation and confidence in the results.

### Use of AI‐assisted writing tools

ChatGPT (GPT‐4, OpenAI) was employed as a language editing tool to improve grammar, coherence, and scientific tone during manuscript revision. All AI‐assisted suggestions were reviewed and validated by the authors to ensure accuracy and scientific integrity. The tool was used solely for language refinement and did not impact data analysis, interpretation, or conclusions.

## RESULTS

The literature search across the PubMed, Embase and Scopus databases yielded a total of 457 records (Embase: 192, Scopus: 175, PubMed: 90). After removing duplicates, 222 records remained for title and abstract screening. Of these, 186 were excluded for not meeting the inclusion criteria, and 36 full‐text articles were assessed for eligibility. During the full‐text evaluation stage, 22 studies were excluded due to insufficient or unavailable data, and one study was excluded because it was a secondary publication of a previously included dataset. Ultimately, 11 studies met all inclusion criteria and were included in the systematic review.

**Figure 2:**
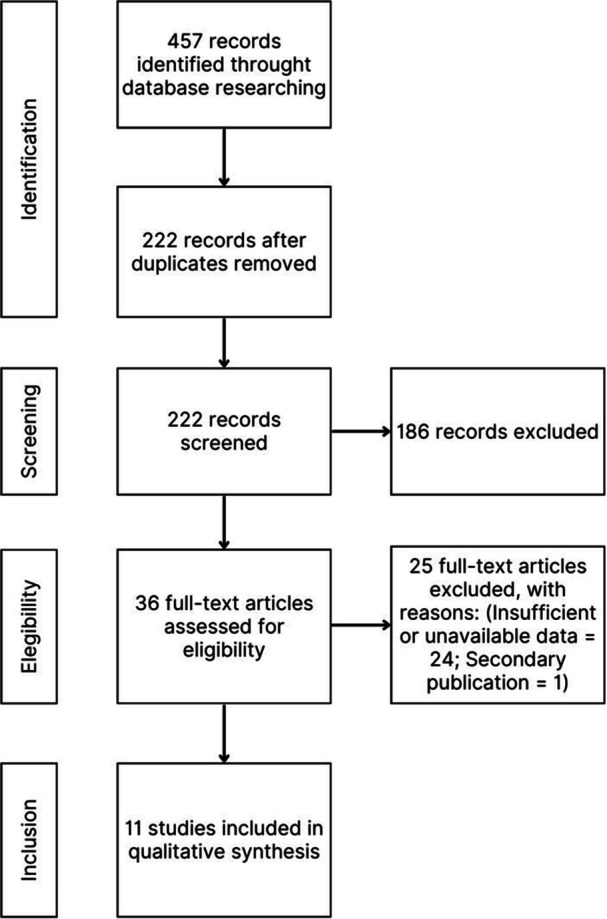
Flowchart of the study selection process. PRISMA diagram detailing the systematic review process, including record identification, screening, eligibility assessment and final inclusion, with reasons for exclusions. PRISMA, preferred reporting items for systematic reviews and meta‐analyses.

Additionally, one potentially relevant comparative study was identified, published only as a conference abstract in 2025. Although it presents promising results, it was not included in the primary analysis due to a lack of a complete, peer‐reviewed publication. No eligible studies were classified as ‘unavailable for retrieval’, and no ongoing trials or associated reports were identified. This is a novel review and does not represent an update of a previous systematic review (Figure 2).

### Risk of bias assessment

The risk of bias was assessed according to the study design, using the Cochrane RoB 2 tool for randomised trials, the ROBINS‐I tool for nonrandomised studies, and the NIH quality assessment tool for case series.

**Figure 3:**
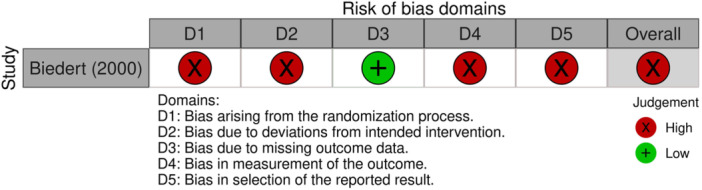
Risk of bias assessment for randomised controlled trials using RoB 2 tool. Summary of the risk of bias evaluation for the randomised controlled trial included in this review, using the Cochrane Risk of Bias 2 (RoB 2) tool.

RCTs (Cochrane RoB 2): [24] The study by Biedert is at high overall risk of bias across several domains. Its randomisation process was inadequate, as patients were assigned by birth date, compromising allocation concealment [2]. Blinding of participants and the surgeon was absent, leading to potential performance bias and subjective outcome measurement by the unblinded surgeon. Additionally, unbalanced deviations from intended interventions occurred, and the reported results were selectively chosen based on statistical significance (Figure 3).

**Figure 4:**
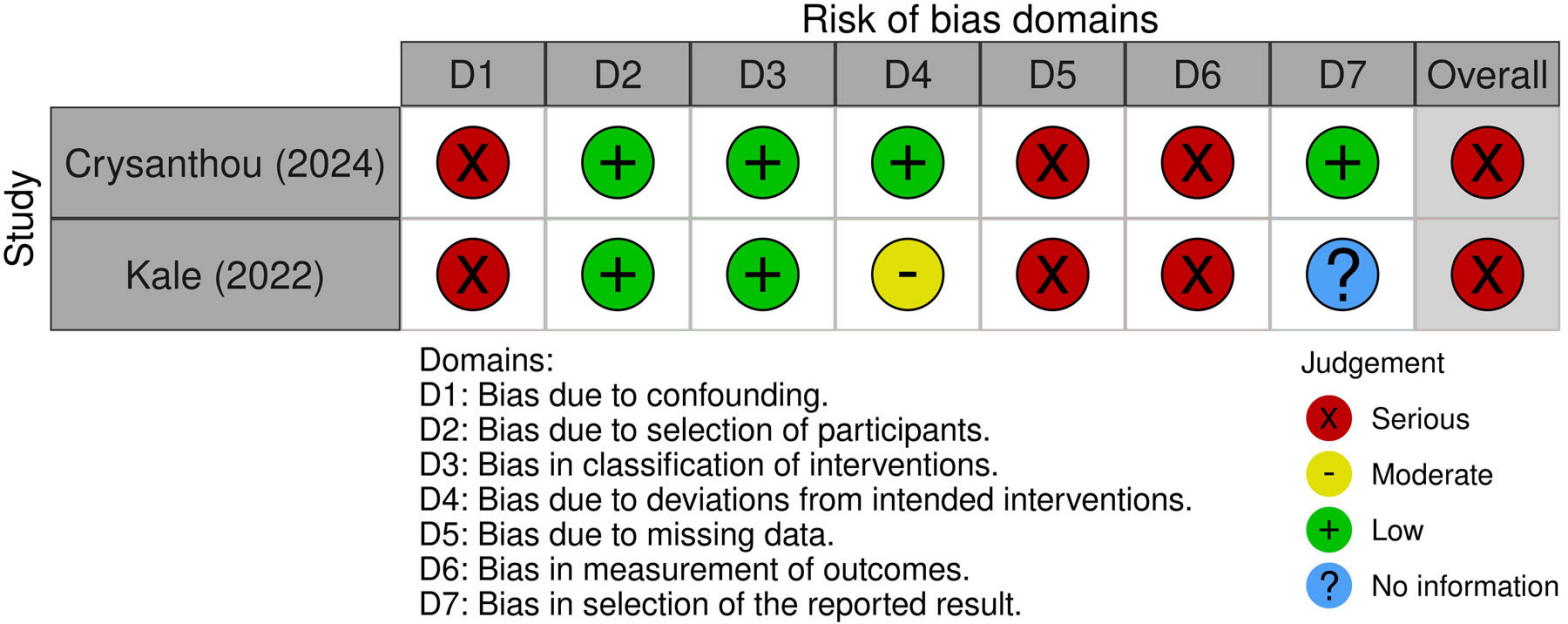
Risk of bias assessment for nonrandomised studies using ROBINS‐I tool. Risk of bias evaluation for the nonrandomised studies included in this review, based on the ROBINS‐I tool (risk of bias in nonrandomised studies of interventions).

Nonrandomised studies (ROBINS‐I): [25] Both the Chrysanthou and Kale studies are assessed as having a serious overall risk of bias due to critical methodological flaws. The Chrysanthou study, despite its quasi‐randomised design, exhibited serious bias due to confounding, significant missing data before allocation and biased outcome measurement for subjective scores resulting from unblinded assessment [5]. Similarly, the Kale study, a single‐arm prospective cohort, exhibited serious biases due to uncontrolled confounding (absence of a control group), unbalanced co‐interventions (notably, concomitant ACL reconstruction, which significantly affected outcomes) and unblinded assessment of subjective clinical and Lysholm scores (Figure 4) [12].

**Figure 5:**
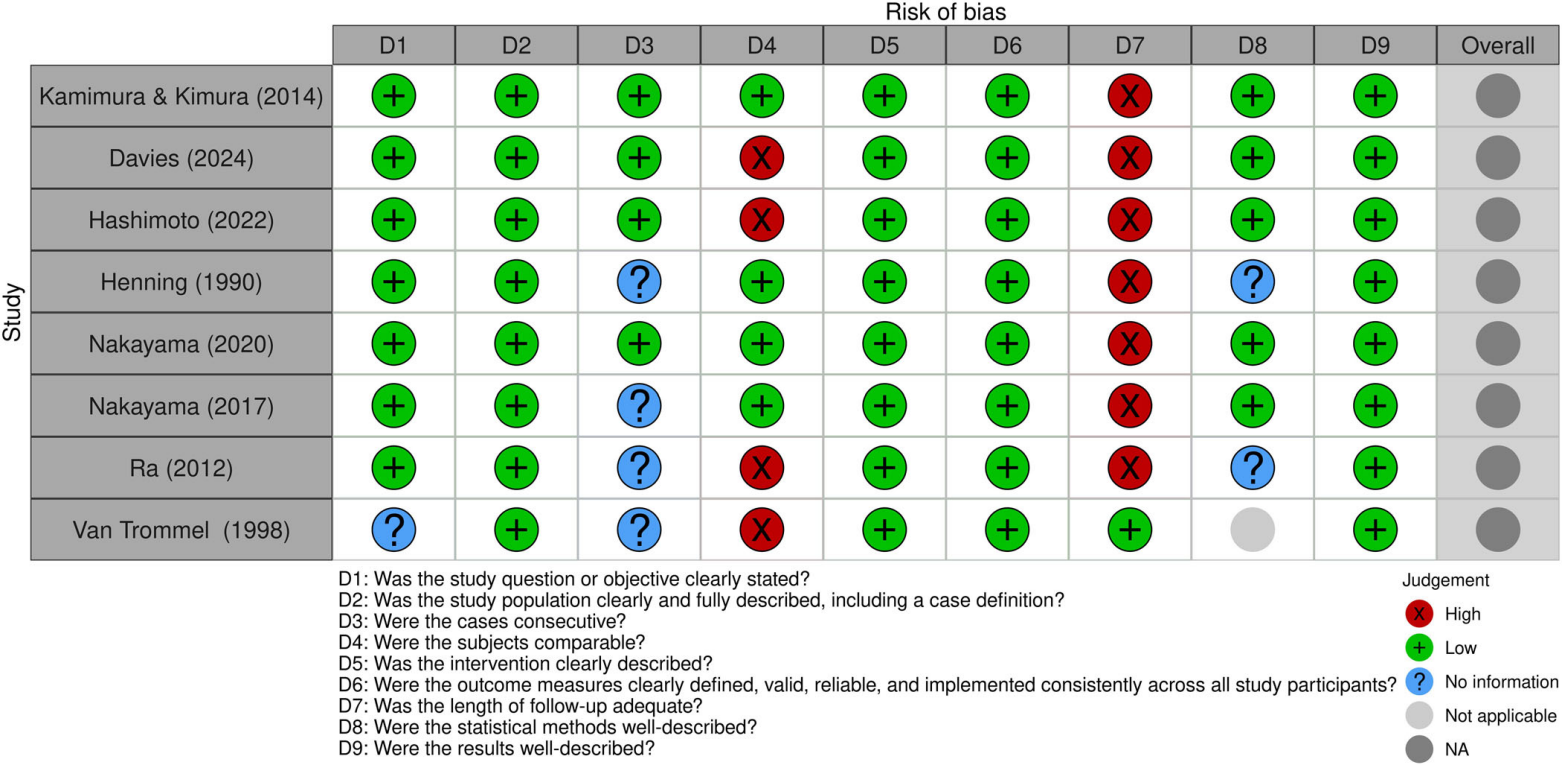
Quality assessment of case series using NIH tool. Summary of methodological quality for case series included in this review, assessed using the National Institutes of Health (NIH) quality assessment tool for case series studies. The NIH tool for case series does not generate an overall risk‐of‐bias score. Instead, it evaluates each criterion individually, allowing for a qualitative judgement rather than a summary rating.

Case series (NIH): [20] Case series studies, by their inherent design, lack a randomised comparison group, making them highly susceptible to various biases (e.g., confounding and selection bias) and fundamentally limiting the ability to infer causality regarding intervention effects. The studies by Kamimura and Kimura; Davies; Hashimoto; Henning; Nakayama et al.; Ra; and Van Trommel consistently demonstrated common methodological limitations [6, 9, 10, 13, 17, 18, 21, 26]. A frequent concern was the inadequate length of follow‐up, which hindered a comprehensive assessment of long‐term outcomes. Many studies also faced issues with subjects not being comparable due to heterogeneity or insufficient description. Furthermore, some studies had unreported consecutiveness of cases and undescribed or inapplicable statistical methods, collectively limiting the trustworthiness and generalisability of their findings (Figure 5).

### Synthesis of the characteristics of included studies

The 11 included studies assessed fibrin clot–augmented meniscal repair techniques across various lesion types, including complete radial tears, degenerative horizontal tears, medial intrasubstance lesions and avascular zone tears. A comprehensive summary of the included studies is provided in Table 1.

**Table 1:** Summary of included studies and main characteristics.

Author (Publication year, country)	Study design	Sample size (ACL injury)	Age (years)	Follow‐up duration (months)	Type of injury	Fibrin clot source	Healing criteria	Healing rate (%)	Failure rate (%)	Clinical scores	Complications	Main findings
Biedert (2000, Switzerland) [2]	Randomised controlled trial	40 Groups: A = 12, B = 10, C = 7, D = 11	30.4 (16–50)	26.5 (12–38)	Degenerative grade II intrasubstance lesions	Commercial fibrin sealant (Tissucol)	Absence of symptoms	43	57	IKDC: 43% normal/nearly normal	Pain, saphenous nerve paraesthesia, and revision surgeries	Partial meniscectomy yielded the best outcomes, and fibrin clot was less effective
Chrysanthou (2024, Greece) [5]	Quasi‐randomised controlled trial	51 Group A = 27, Group B = 24	28.2 ± 6 (17–40)	3 and 12	Meniscal tears (various types)	Autologous peripheral blood	MRI signal normalisation	91.7	8.3	Lysholm: 63 ± 4 to 95 ± 5	No severe adverse events	Higher rates of complete healing in the fibrin clot group (91.67% vs. 70.37%); significant improvement in functional scores
Davies (2024, Australia) [6]	Retrospective case series	50	34 (14–70)	46 (22–87)	Meniscal tears (various types)	Autologous peripheral blood	Absence of symptoms	90.2	9.8	Lysholm: 59 ± 21 to 95 ± 10	1 patient with stiffness (resolved), 1 with altered sensation (improving	Significant improvement in PROMs; reliable and reproducible technique with minimal morbidity
Hashimoto (2021, Japan) [9]	Retrospective case series	30	23.2 ± 9.6 (13–46)	25.92 (24–48)	Meniscal tears in the avascular zone	Autologous bone marrow aspirate	MRI signal normalisation	93.3	6.7	Lysholm: 65.1 ± 11.7 to 95.6 ± 6.8	No severe adverse events	Significant improvement in Lysholm scores; enhanced healing with minimal complications
Henning (1990, USA) [10]	Retrospective case series	153 (40)	23 (14–45)	2 months to several years^a^	Unstable meniscal tears (various types)	Autologous peripheral blood	Absence of symptoms	92	8	Not reported	Retear, neurovascular injury	Autologous fibrin clot improves healing for young athletes
Kale (2023, India) [12]	Prospective cohort	30 (16)	Not specified (within the age limit of 60)	24	Meniscal tears (various types)	Autologous peripheral blood	Absence of symptoms	96.6	3.4	Lysholm: 67.63 ± 6.55 to 92.0 ± 2.9	2 cases of paraesthesia, 1 case of recurrence	Significant improvement in Lysholm scores 96.6% clinical healing
Kamimura and Kimura (2014, Japan) [13]	Prospective case series	10	36.5 ± 12.5	40.8 ± 5.4 (32.5–51.3)	Degenerative horizontal meniscal tears	Autologous peripheral blood	Arthroscopy complete healing	70	30	IKDC: 26.5 ± 19% to 87.8 ± 7.5%; Lysholm: 69.3 ± 16.3 to 95.4 ± 3.6	No severe adverse events	Significant improvement in functional scores; 70% of cases achieved complete healing, but partial healing in 30%
Nakayama (2017, Japan) [17]	Retrospective case series	46	22.9 ± 9.6 (12–50)	19.8 ± 6.8 (12–33)	Meniscal tears in the avascular zone	Autologous peripheral blood	Absence of symptoms	91.3	8.7	Lysholm: 74 ± 14.3 to 98.3 ± 2.2	No severe adverse events	80% of athletes returned to sports; high healing rates with fibrin clot in avascular regions
Nakayama (2020, Japan) [18]	Retrospective case series	24	47.0 ± 8.1 (35–66)	39.3 ± 11.6 (24–64)	Degenerative medial meniscal tears	Autologous peripheral blood	Absence of symptoms	75	25	Lysholm: 75.4 ± 4.6 to 90.8 ± 5.7	No severe adverse events	75% success rate in clinical healing; varus alignment was a significant risk factor for repair failure
Ra (2013, South Korea) [21]	Retrospective case series	12 (2)	Not specified	30 ± 4	Complete radial tears	Autologous peripheral blood	MRI signal normalisation	91.6	8.4	IKDC: 57 ± 7% to 92 ± 3%; Lysholm: 65 ± 6 to 94 ± 3	No severe adverse events	Significant improvement in functional scores; 11 of 12 cases showed complete healing on MRI
Van Trommel (1998, USA) [26]	Retrospective case series	5 (1)	20 (18–22)	71 (66–81)	Complete radial tears in the avascular zone	Autologous peripheral blood	Arthroscopy complete healing	60	40	Not reported	No severe adverse events	Effective for avascular portion healing, all returned to sports

*Note*: This table summarises the included studies, highlighting methodological design, patient profiles, injury characteristics, follow‐up duration and functional and healing outcomes. Data are presented as mean ± standard deviation or percentage, as reported in each study. When PROMs were reported at multiple time points, preoperative and final follow‐up values are presented. Abbreviations: ACL, anterior cruciate ligament; IKDC, International Knee Documentation Committee; MRI, magnetic resonance imaging; PROM, patient‐reported outcome measures.

a No single mean reported for all cases; follow‐up varied from 2 months to several years after injury. Some specific subsets had follow‐up at 4 months postoperatively

### Fibrin clot preparation and application techniques

The most common preparation method involved drawing venous blood intraoperatively (50–60 mL) and allowing it to coagulate spontaneously in a sterile syringe or container for 5–15 min [5]. The formed clot was then shaped and handled using sterile instruments before being inserted into the lesion. Hashimoto et al. used clots derived from bone marrow aspirate, typically obtained from the iliac crest or tibia [9].

Application techniques varied and were grouped into five main strategies:

1. **Inside‐out suture techniques with clot insertion during fixation**, as described by Ra et al.; Van Trommel et al.; Henning et al.; and Davies et al. In these studies, the inside‐out sutures are passed through the meniscus in the usual fashion. The fibrin clot is then positioned between the torn meniscal edges before tying the sutures, allowing the suture loop itself to compress and secure the clot. An extra suture may be added if needed [6, 10, 21, 26].
2. **Manual clot insertion using instruments (e.g., hemostat), followed by inside‐out suturing**, was employed by Kale et al. In this technique, the clot is first positioned in the lesion using a hemostat, which allows for more precise control over its placement. Once the clot is situated correctly, the inside‐out sutures are then passed and tied, securing the meniscal edges over the clot [12].
3. **Direct clot insertion**, as used by Biedert; Kamimura and Kimura; and Nakayama et al., differ from inside‐out‐based techniques. Here, the clot is manually inserted into the lesion cavity, and then sutures are applied using vertical or horizontal techniques, which may be inside‐out or outside‐in. The clot acts as a filler or scaffold, and the repair technique is adapted to compress the meniscal tissue around it [2, 13, 17, 18].
4. **Circumferential suturing with inside‐out insertion**, as described by Hashimoto et al., involves placing a bone marrow–derived clot into the lesion using an inside‐out approach, followed by securing it with circumferential sutures, which may provide both biological integration and enhanced mechanical stability [9].
5. **Suture shuttling technique**, described by Chrysanthou et al., involves attaching absorbable sutures to each end of a premoulded fibrin clot, which is then pulled into the tear using arthroscopic instruments. Once inside the lesion, the sutures are tied, securing the clot in place. This method enables precise and controlled delivery, particularly in complex tear configurations [5].

The choice of technique varied across studies and was often tailored to tear type, meniscal zone and surgeon preference. Despite procedural variability, most techniques aimed to maximise clot integration and stability within the repair site.

### Complications and safety of the technique

The technique demonstrated a favourable safety profile, with consistently low complication rates across the included studies. No significant adverse events were directly attributed to the use of the fibrin clot. Reported failures were predominantly related to the persistence or recurrence of symptoms, rather than complications arising from the procedure itself. When present, minor complications were primarily associated with the arthroscopic procedure or the suture technique, rather than the fibrin clot [6, 12, 13, 17, 26].

For example, Biedert reported cases of saphenous nerve paraesthesia and postoperative pain; Kale et al. described two cases of paraesthesia and one case of recurrence that required partial meniscectomy [2, 12]. Davies et al. noted one case of transient joint stiffness and one of altered sensation, both of which resolved or improved with conservative treatment [6]. These findings reinforce the conclusion that fibrin clot augmentation is a safe and well‐tolerated technique.

## DISCUSSION

This systematic review aimed to evaluate the role of the fibrin clot as a biological adjunct to meniscal repair. Although the technique has been employed for decades and is biologically plausible, the current clinical evidence remains inconclusive, predominantly based on studies of low methodological quality.

Most studies are retrospective case series, often cited as Level IV evidence. This study design inherently carries a higher risk of bias compared to higher‐level evidence studies, such as RCTs, as it often lacks control groups and randomisation, which are crucial for minimising confounding factors. Only one study deviated from this: Biedert conducted a randomised prospective study comparing four different treatment methods, including fibrin clot, partial meniscectomy and conservative therapy. Patients were ‘randomly assigned by birth date’ which, while a form of randomisation, might not be as robust as accurate random allocation [2].

A primary concern was the absence of a control group in most studies, which was explicitly acknowledged as a limitation by authors such as Kamimura and Kimura; Hashimoto et al.; Nakayama et al.; Ra et al. and Kale et al. [9, 12, 13, 18, 21]. Without a comparator—whether it be partial meniscectomy, meniscal repair without fibrin clot, or no treatment—it is difficult to determine whether the observed outcomes can be explicitly attributed to the use of fibrin clot augmentation or other confounding variables, including the natural healing process.

Another standard limitation was the small sample size reported in most studies. For example, Kamimura and Kimura included only 10 patients, Van Trommel et al. reported on five cases, and Nakayama et al.; Hashimoto et al. and Davies et al. analysed cohorts of 24, 30 and 50 patients, respectively [6, 9, 13, 18, 26]. These limited sample sizes reduce the statistical power of the findings and limit the generalisability of the results to broader patient populations.

The follow‐up periods were also generally short, which hinders the ability to assess long‐term outcomes such as sustained meniscal healing, development of osteoarthritis, or late surgical failures. This limitation was acknowledged in multiple studies, including those by Davies et al.; Hashimoto et al. and Nakayama et al. [6, 9, 17, 18]. Some authors noted that failure rates may have been underestimated due to insufficient follow‐up duration.

Outcome assessment methods introduced additional sources of bias. Most studies relied heavily on subjective clinical scores, such as the Lysholm and IKDC scores, as well as MRI findings, without a consistent use of second‐look arthroscopy to confirm healing. This reliance on indirect measures can present a ‘best‐case scenario’ and may underestimate the actual rate of repair failure [9]. Moreover, MRI interpretation postoperatively is known to be challenging, as scar tissue and fibrin clots can mimic meniscal tears for several months after surgery [2, 6]. In several cases, outcome evaluations were conducted by the operating surgeon, raising the risk of observer bias [17].

There was also considerable heterogeneity in the patient populations and tear characteristics included in the studies. Differences in patient age, type and location of meniscal tear and associated conditions (e.g., discoid menisci, as seen in Kamimura and Kimura) complicate the extrapolation of results to specific subgroups [13]. Several studies were retrospective in design, which carries inherent risks of selection bias and confounding [6, 9, 10, 17, 18, 21, 26]. The use of retrospective data can result in incomplete or inconsistent documentation, such as missing preoperative PROMs or unmeasured variables like limb alignment, potentially impacting outcome interpretation [6, 9].

Moreover, including concomitant ACL injuries in meniscal repair studies introduces a significant risk of bias, primarily due to confounding and potential performance bias. The presence and subsequent reconstruction of an ACL injury fundamentally alter knee stability, vascularity and postoperative rehabilitation, creating a distinct biomechanical and biological environment for meniscal healing [6, 13]. Many studies explicitly exclude patients with concomitant ACL reconstruction to maintain sample homogeneity and minimise these confounding factors [6, 13, 17, 18]. Failure to adequately account for or exclude these complex cases can lead to biased outcome interpretations, as the ACL injury and its treatment can independently influence meniscal repair success and patient outcomes [12, 17].

Lastly, performance bias was noted in studies where a single, highly experienced surgeon conducted all procedures. While this may ensure technical consistency, it also limits the generalisability of findings to less experienced surgeons or varied clinical environments. Davies et al. directly addressed this issue, noting that the senior surgeon′s extensive experience may not reflect typical surgical practice elsewhere [6].

Despite these limitations, several studies reported high clinical success rates. The findings suggest that fibrin clot augmentation is particularly effective for treating complex tears in the avascular zone, where reported outcomes have consistently been favourable. Studies by Ra and Hashimoto demonstrated healing rates of 91.6% and 93.3%, respectively [9, 21]. These results are promising, particularly when compared to historical data where standard repair techniques in avascular zones were associated with failure rates of 20%–40% [7, 23].

In the study by Van Trommel et al., although two out of five patients required partial resection of the repair site during second‐look arthroscopy due to incomplete healing, the authors considered the repair successful if the peripheral rim had healed adequately, given its critical role in restoring hoop tension and distributing load across the knee [26]. Based on this criterion, all repairs were classified as successful in achieving functional reinforcement of the meniscus. The authors note that the posterolateral aspect of the lateral meniscus adjacent to the popliteus tendon is devoid of penetrating peripheral vessels, making it challenging to heal [26]. A complete radial split at this site is typically treated with total meniscectomy. Enhancing the repair with a fibrin clot may represent a potential method for achieving good results in this challenging avascular zone [26].

In contrast, degenerative meniscal lesions demonstrated more variable outcomes, as reported in studies by Nakayama et al. and Kamimura andKimura. In Kamimura and Kimura′s study, follow‐up MRI revealed approximately 30% of cases with incomplete healing [13]. In the 2020 cohort by Nakayama et al., the reported healing rate was around 75%, although no imaging confirmation was provided [18]. These variations may reflect the intrinsic structural degeneration and reduced cellular viability typically seen in degenerative meniscal tissue, which could limit the regenerative potential of fibrin clots despite their biological activity. Notably, Nakayama et al. identified varus malalignment in 100% of the knees in the repair failure group, suggesting that lower limb alignment may be an important prognostic factor influencing healing outcomes in degenerative meniscal repairs [18].

As for the type of fibrin clot employed, the majority of included studies used autologous clots, derived from peripheral blood or bone marrow aspirate. These formulations are believed to provide a biologically active scaffold rich in growth factors and cytokines, potentially enhancing healing in avascular or complex tear patterns [9]. In contrast, only one study among those included—Biedert—used a commercial fibrin sealant (Tissucol®) [2]. In that randomised trial focused on degenerative grade II intrasubstance lesions, the use of the commercial clot was associated with the worst clinical outcomes among the treatment groups, including high rates of persistent symptoms and a low percentage of normal or nearly normal IKDC scores [2]. These findings suggest that synthetic formulations may lack the cellular and molecular components necessary for optimal integration and healing.

Another relevant study, employing a commercial fibrin glue (Tisseel), was conducted by Ishimura et al. [11]. Although this study was not included in the present systematic review—as it comprised patients treated without meniscal suturing and did not report outcomes separately for glue‐only versus suture‐based repairs—it offers valuable comparative insight. Ishimura′s cohort consisted predominantly of traumatic meniscal tears, including longitudinal and bucket‐handle lesions, many located in the red‐red vascular zone, and frequently associated with ACL injuries. Over a long‐term follow‐up (mean 8.2 years), the authors reported favourable clinical and arthroscopic outcomes, with a recurrence rate of only 10% [11].

The contrast between Ishimura′s positive results and Biedert′s unfavourable outcomes likely reflects key differences in lesion type (traumatic vs. degenerative), vascularity of the tear location, and patient profile, rather than the efficacy of the commercial clot per se [2, 11]. Furthermore, the lack of standardised suturing in Ishimura′s protocol introduces additional limitations to direct comparison [11]. Nevertheless, both studies emphasise the importance of contextualising the use of fibrin sealants within the biological and mechanical environment of the lesion. These findings reinforce that outcomes with fibrin‐based augmentation depend not only on the source of the clot—autologous or commercial—but also on the tear characteristics, repair technique and associated joint stability.

Compared to other biological augmentation strategies, such as platelet‐rich plasma (PRP), scaffolds and marrow stimulation, fibrin clot augmentation is notable for its accessibility, cost‐effectiveness and safety profile. Although PRP has demonstrated potential in reducing failure rates and alleviating pain in some studies, it requires centrifugation equipment and incurs higher costs [15, 28]. Scaffolds offer biomechanical advantages but are expensive, technically complex and associated with moderate complication rates [8]. Marrow stimulation presents another cost‐effective option and has shown improved healing outcomes in controlled trials [3, 14].

Our findings align with prior systematic reviews, such as those by Zaffagnini et al., which recognised the biological rationale for meniscal repair augmentation but concluded that the available clinical evidence was insufficient to support its routine use [28]. Their review evaluated multiple biologic strategies—including PRP, fibrin clot and mesenchymal stem cells—but included only two studies specifically addressing fibrin clot, both of which lacked control groups. In contrast, the present review offers a more comprehensive synthesis, incorporating eleven studies that focus exclusively on fibrin clots, covering diverse tear patterns, meniscal zones and application methods. This broader and more targeted analysis provides a clearer understanding of the clinical contexts in which the fibrin clot may offer potential benefits.

### Limitations and future directions

Although several studies reported promising results, the current body of evidence remains insufficient to establish the clinical effectiveness of fibrin clot as an adjunct to meniscal repair. Most available data come from small, uncontrolled case series with heterogeneous populations, tear patterns, surgical techniques and outcome assessments. The lack of standardised definitions for healing and failure, limited use of imaging or arthroscopic confirmation and short‐to‐intermediate follow‐up durations further limit the strength of the conclusions.

Despite its low cost, favourable safety profile and biological rationale—particularly for avascular or complex tear patterns—fibrin clot augmentation cannot yet be recommended for routine use in clinical practice. Its application may still be considered on a case‐by‐case basis, especially in situations where intrinsic healing capacity is limited.

To clarify the actual value of this technique, future research should focus on well‐designed RCTs comparing fibrin clot–augmented repairs with standard methods. These studies should include clear inclusion criteria, standardised surgical protocols, validated outcome measures and long‐term follow‐up. Comparative studies evaluating fibrin clot against other biologic adjuncts, such as PRP, bone marrow aspirate concentrate, or marrow stimulation, would also contribute to a more evidence‐based approach to biologic enhancement in meniscal preservation.

## CONCLUSION

The use of autologous fibrin clot as a biologic adjunct to meniscal repair is conceptually attractive and technically feasible. However, current clinical evidence is limited and methodologically weak. Although some studies suggest improvements in healing rates and functional outcomes, the absence of rigorous comparative data and the high risk of bias across most studies preclude definitive conclusions.

Currently, there is insufficient evidence to support or refute the routine use of fibrin clot in meniscal repair. Its role should be further investigated through high‐quality prospective studies designed to assess its efficacy across different tear patterns and patient populations. Until such data are available, their use should remain selective and guided by clinical judgement rather than routine protocol.

## AUTHOR CONTRIBUTIONS

Laura Amaral Coelho de Azevedo and Rayanne Carneiro Torres de Novaes performed the study selection. Laura Amaral Coelho de Azevedo and Gabriela de Paula Leite Rocha Alcântara Del Campo extracted the data. Disagreements during the selection or extraction process were resolved by Pedro Baches Jorge and Diego Escudeiro de Oliveira. All authors contributed to study design, data interpretation, manuscript writing and approved the final version.

## CONFLICT OF INTEREST STATEMENT

The authors declare no conflicts of interest.

## ETHICS STATEMENT

No ethical approval was required for this systematic review.

## Data Availability

Data sharing is not applicable to this article as no datasets were generated or analysed during the current study.
